# Daily Optional Physical Education Does Not Counteract Increasing Inactivity by Age among Adolescents

**DOI:** 10.3390/children10121929

**Published:** 2023-12-14

**Authors:** Zsuzsa Lábiscsák-Erdélyi, Annamária Somhegyi, Ilona Veres-Balajti, Karolina Kósa

**Affiliations:** 1 Department of Physiotherapy, Faculty of Health Sciences, University of Debrecen, 4028 Debrecen, Hungary; balajti.ilona@etk.unideb.hu; 2 Schools for Health in Europe Network Foundation, National Center for Spinal Disorders, 1126 Budapest, Hungary; annamaria.somhegyi@bhc.hu; 3 Department of Behavioral Sciences, Faculty of Medicine, University of Debrecen, 4032 Debrecen, Hungary; kosa.karolina@med.unideb.hu

**Keywords:** physical education, healthpromoting schools, short-term evaluation

## Abstract

Background: This paper describes the outcomes of an integrated health promotion programme implemented in a Hungarian high school offering health education in the curriculum, daily optional physical education, teacher training in applying a person-centered approach in teaching, and parental involvement in school activities. Methods: The evaluation used mixed methods of which results of the before-6-months-after quantitative survey among pupils is described. The health status and behaviour of students were assessed by applying the Hungarian version of the HBSC questionnaire. Results: Significant improvement was found in the self-rated health of girls (6.6% increase in being of excellent health, *p* = 0.04), and the consumption of sweets and sugary soft drinks decreased significantly for both genders (boys: −10.2%, *p* = 0.01; girls: −6.06%, *p* = 0.04). However, the proportion of physically inactive girls significantly increased (girls: 11.2%, *p* = 0.01), and substance use did not change significantly. Discussion and conclusions: The intervention had significant positive impacts on subjective health and dietary habits and could counteract the secular trend of increasing tobacco, alcohol, and drug consumption by age among adolescents, but this unfortunately does not include physical inactivity. Offsetting the most widespread health risk behavior, physical inactivity, may require mandatory daily physical education in schools.

## 1. Introduction

The concept of school health promotion (HP) was initiated by the World Health Organization (WHO) in 1995 [1]. Its holistic model of health suggested actions to improve the biological, psychological, and social factors conducive to a healthier life not only for students, but also for teachers, school workers, and parents alike. The concept emphasised the contextualisation of actions to address local needs and conditions, building on the experiences of the European Network of Health Promoting Schools (ENHPS), which was founded in 1991 [2].

One of the major challenges of health promotion actions in general, and school health-promoting projects in particular, has been their evaluation, which was the topic of several international workshops—the first in 1998—and a number of publications [2,3,4]. The evaluation of school health programmes provides vital information for improving the quality and effectiveness of future interventions, but it is much less straightforward in real-life settings than the evaluation of medical or educational interventions because of several issues. Health-promoting interventions in schools are almost always quasi-experimental. In these programmes, the unit of analysis, sampling, the separation of secular trends from the impact of the programme, and the reliability of statistical results must all be carefully considered. Due to the lack of well-founded theories, conclusions about complex cause–effect relationships have considerable limits [5].

The recommended design of evaluation should aim for participatory, involving stakeholders in the programme; should use mixed, qualitative, and quantitative methods as well; should be realistic in terms of understanding the mechanisms by which the interventions produce changes; and should also uncover the contextual conditions in which changes take place [6,7,8,9].

Large numbers of programmes were implemented all over the world since the introduction of the concept of school HP in 1995, but we found only 23 scientific papers on the evaluation of integrated school health promotion programmes published in English. The majority of these papers gave accounts of short-term evaluations; two articles were identified with at least two years of follow up. A programme in Portugal [10] found large improvements in social and emotional skills of elementary school children in a 4-year follow-up study. A Dutch programme evaluated the health behaviour, anthropomorphic and psychosocial features of students for two years after the implementation of a health-promoting intervention in two schools and found a significant reduction in the body mass index and reduced screen time in one school, but not in the other [11].

Our paper describes the short-term follow up of a school health-promoting programme, shortly after which legislation was introduced to expand health education and promotion in public schools in Hungary. The concept of school health promotion was spread in the country by the Association of Hungarian Healthy School Network established in the mid-1990s. A complex health promotion programme in Pécs was shown to be effective in a domestic evaluation report [12]. Health education and health protection were integrated into the curriculum by a decree of the Ministry of Education in 2000 [13], amended in 2003, but comprehensive health-promoting programmes in schools remained sporadic. Local school projects were funded in the framework of the New Hungary Development Plan (SROP 6.1.2/A/09/1), financed by the European Social Fund and the Government of Hungary. One project was financed and granted to a high school in Debrecen in 2009, enabling the implementation of a health-promoting project between April and July 2011. The programme placed special emphasis on the uptake of daily optional physical education that was implemented to meet the international guidelines that school-aged children should accumulate at least 60 min of physical activity daily [14]. The present paper describes the short-term evaluation of this project carried out 6 months after the baseline survey. Due to financial limitations, limited availability of human resources, and taking into consideration the viewpoints of a major stakeholder, the faculty of the school, our evaluation focused on students by applying quantitative methodology. In order to compare the school data to the national data, the methodology followed the Health Behaviour in School-aged Children (HBSC) [15].

## 2. Materials and Methods

### 2.1. Description of the Intervention in Alignment with the WHO Health-Promoting School Framework [16]: The School Health Promotion Programme Had Four Main Components

1. Implementation of healthy nutrition in order to improve the quality of school meals.
2. Promotion of daily physical activity for all students via joint physical activity programmes (11 programmes between April and September 2011).
3. Adoption of teaching methods (teacher training) that improve students’ health:
  - Person-centred pedagogical methods (four senior teachers follow a supervision course (30 h); 33 + 7 teachers follow a case-based course (5 × 6 h));
  - Implementing arts (course for two teachers);
  - Short course in gymnastics for five teachers;
  - Long course in gymnastics for one PE teacher;
  - Renewal of dance pedagogy training for one dance and drama teacher;
  - Development of interpersonal skills for 16 teachers;
  - Relaxation training for seven teachers.
4. Health education, with particular attention to topics of particular interest to students. These thematic projects directly or indirectly targeted not only the students, but also parents, teachers, and all school staff. The implementation of the health promotion programme was facilitated via the preparedness of the teaching staff and the harmonious cooperation with the school health service, parents, and NGOs associated with the school throughout the programme.

### 2.2. Study Design

A pre-test–post-test study design was applied by implementing repeated cross-sectional surveys to evaluate the impact of a health-promoting programme implemented in the Spring semester of the school year of 2010/2011. A comprehensive baseline survey of the students was carried out before the start of the programme, and the first evaluation survey occurred 4 months later, in the Fall semester of the next school year. The analysis was restricted to pupils in those study years who received the intervention and had been in school at the time of evaluation, in the next school year. A short-term evaluation of the programme was carried out by comparing the data of students in 9th grade, 10th grade, and 11th grade at the baseline survey with those of 10th grade, 11th grade, and 12th grade students at the follow-up survey. We hypothesised that students’ health behaviour favourably differed after the health promotion programme compared to before.

### 2.3. Variables

Items of the baseline questionnaire had been taken from the Hungarian version [17] of the Health Behaviour of School-aged Children (HBSC) 2010 survey [18]. Demographic data included gender, date of birth, and school grade. Socioeconomic status indicators, such as parents’ highest education (higher education diploma, high school graduation, vocational school, completed primary school, or less than primary school), type of permanent place of residence (county seat, city, village, or farm), and number of computers in the household (none, one, two, or more than two) were used to create a composite indicator with a range of 4–18. This indicator was used to check the reliability of perceived family wealth. Regarding health behaviour, physical activity (PA) was assessed via three questions: the number of PA occasions in leisure time during which they were active to the point of sweating, the number of hours per week out of school during which they were active to the point of sweating, and the number of days of participating in physical activity classes in school (which was available every school day in this school, as opposed to other schools). In order to assess physical activity, a composite variable from all three PA variables was created with three categories: PA was categorised as moderate-to-vigorous if the pupil had PA at least 4 times and 4 h per week in leisure time and attended PA class in school every day; PA was moderate if the pupil had PA 2–3 times and 2–3 h in leisure time and attended PA class in school at least 3 days but not every day per week, and pupils were classified inactive if they had PA less than 2 times and less than 2 h per week in leisure time and attended PA class in school less than 3 days per week. 

As for dietary habits, the frequency of purchasing unhealthy snacks such as chocolate, chips, sugary drinks, and sweets in the school canteen were reported on a 3-point response scale (frequently, sometimes, or never) from which a composite variable was created to assess the consumption of these snacks. The resulting binary variable had a value of 0 if the responder never consumed any of these four snacks, and a value of 1 signified all other responses. Consumption of breakfast and lunch on school days were answered on a 6-point scale, both of which were collapsed into three categories (every day, some days, or never). Tobacco use was reported as daily use, more than once a week, weekly, or non-smoker. The lifetime prevalence of drunkenness was reported in five categories from “never” to “more than 10 times”. Drug consumption was assessed via six items (ecstasy, speed, alcohol with medicine, medicine, solvents, or cannabis), with each answerable on a 7-point scale ranging from “never” to “more than 40 times”, from which a composite variable was created and collapsed into a binary variable with a value of 0 if none of these drugs were consumed and a value of 1 for all other responses. Leisure time activities of watching television or video and using a computer for various activities were assessed via two questions answerable on a 9-point scale ranging from “no time” to “7 h or more” and are reported as two categories (no more than 2 h, 3 h, or more). Self-rated health was assessed via one item answered on a 4-point scale (bad, average, good, or excellent). 

### 2.4. Data Collection

A web-based questionnaire was developed for data collection. The network server was established by an IT officer working with the research team. A standard Linux server was used with PHP and MySQL support. The questionnaire contained a brief description at the top of the website informing the students about the purpose and design of the study, followed by items of the questionnaire. Participants were asked to provide an identifier of their choice (up to 10 characters), so their internet protocol (IP) address was not incorporated in the database. The pre-trial tests showed that the questionnaire can be completed in 20 min. Access to the questionnaire was pre-organised in a scheduled time point for groups of participating students in the computer room of the school. The test was not available outside of the scheduled times. The same questionnaire was used in the baseline survey before the intervention, and in the follow-up survey to make the surveys fully comparable. The ethical permission was issued by the Regional Research Ethics Committee of the University of Debrecen (DE OEC RKEB/IKEB 3475-2011).

### 2.5. Data Analysis

Data were automatically logged in a database, upon completion of the questionnaire, from which the data were downloaded in a Microsoft Excel file. Records were checked for duplicates, empty records, and answers out of the specified ranges, which were removed from the database. After cleaning, data analysis was carried out in STATA 13.0. Gender differences were checked and when found, the data for boys and girls were analysed separately. Continuous variables were compared via *t*-test and categorical variables were analysed via the chi-square test, after checking for the appropriateness of the test conditions. The level of significance was set at 0.05.

## 3. Results

### 3.1. Description of the Family Background of Students

The site of the implementation was one high school in the second largest city of the country. The socioeconomic background of the families of students in this school was compared to the national data in the 2010 Hungarian report of the HBSC. The data for students in grades 9 and 11 in the national report could not be separately analysed, so characteristics of the full Hungarian HBSC cohort of 5-7-9-11 grades in 2010 was compared to students in the intervention school. The data of students in all study years in the intervention school was used for this comparison, which shows a significantly higher proportion of girls, a lower proportion of city dwellers, a larger proportion of both parents with higher educational qualifications, and a higher number of computers in the homes of students in this school compared to those in the national survey (Table 1). 

Perceived family wealth was significantly different in the samples, revealing that more than one-third of students in the national HBSC sample lived in at least quite well-off families compared to slightly more than one-quarter of students in the intervention school. To check the reliability of this indicator, a composite indicator for socioeconomic status was created using the type of permanent place of residence, the parents’ educational status, and the number of computers in the household as described in the Materials and Methods section.

### 3.2. Socio-Demographic Data of the Participating Students

The total number of students in the high school was 1141 in April 2011. A total of 947 students filled the questionnaire and 944 records were eligible for analysis after cleaning. Of those, 747 students in grades 9, 10, or 11 were included in the baseline survey (mean age 16.7 ± 0.92 years; 59.5% female). Grade 12 were omitted because they would leave the school at the end of the school year and would not be available for the first evaluation that was carried out at the beginning of the next school year (next Fall). The evaluation at Fall was carried out among students in grades 10–12 (who were in grades 9–11 at the previous semester, during the intervention), of whom 687 students completed the questionnaire. Girls comprised the majority of the students in all grades. The number of students in the surveys and their gender distribution are shown in Table 2. 

### 3.3. Health Behaviour

#### 3.3.1. Physical Activity

Physical activity was assessed via three indicators, two of which were related to leisure time, and one to school. No significant change in leisure time physical activity at the follow up compared to the baseline among boys and girls was seen; however, the proportion of students physically active 4–7 times per week decreased by 3.8% (boys) and 3.5% (girls). Regarding the number of leisure hours spent with physical activity per week, it does not change significantly either, but this change occurred for the worse for boys and girls. The proportion of boys spending at least 4 h per week with physical activity decreased by 4.3% and this proportion among girls decreased by 3.5%, as shown in Table 3. 

One variable on physical activity measured participation in a physical education class at school (provided every school day in Hungary). The proportion of boys participating every day decreased by 0.93%; however, the proportion of daily participant girls increased by 1.15%, but neither of these changes were significant. A composite variable was created from the above three items to assess overall physical activity as described in the Methods section. A total of 261 students at the baseline and 249 students at the follow up could be allocated to any of the three categories (moderate-to-vigorous, moderate, or inactive) due to their consistent answers, so the composite indicator could only be created for them. Significant change was observed in girls: the proportion with moderate-to-vigorous activity decreased by 12.25% among girls, *p* = 0.01 (Table 3). 

#### 3.3.2. Dietary Habits

Consumption of unhealthy snacks was assessed via a composite variable created, as described in the Methods section, to capture the consumption of chocolate, chips, sugary drinks, and sweets. This binary variable was used to distinguish between those who never purchased any of these items in the school canteen versus those who did. The proportion of students never having purchased any of these snacks significantly increased from baseline to follow up both among boys (by 10.24%, *p* = 0.01) and girls (by 6.06%, *p* = 0.04), as shown in Table 4.

Consuming breakfast and lunch on school days also changed by the time of the follow up for the better compared to the baseline. A total of 5.29% more boys ate breakfast every school day and the proportion of girls having breakfast every school day also increased by 2.68%, but neither of these were significant. The proportion of pupils eating lunch every school day increased in both genders (by 3.98% in boys, 5.63% in girls; Table 4).

#### 3.3.3. Substance Use

The proportion of non-smokers decreased from baseline to follow up in both genders (boys: −1.09%, girls: −1.92%,), and the proportion of daily smokers rose by 2.65% among boys and by 1.48% among girls. The proportion of those who never got drunk in their life decreased (boys −5.42%, girls −4.10%), and the proportion of those who got drunk more than ten times increased (boys 10.89%, girls 0.58%). The change in the frequency smoking and drunkenness from baseline to follow up was not significant either. Consumption of the six drugs was assessed via a composite variable described in the Methods section. The proportion of those who never consumed any of the six drugs decreased both in boys (−4.66) and girls (−2.45%), but neither of them were significant changes (Table 5).

#### 3.3.4. TV or Computer Use in Leisure Time

A total of 1.80% less boys and 2.11% less girls spent 3 h or more with this activity at the follow up compared to the baseline value, showing non-significant improvement in both genders. Computer time also decreased in both genders with a larger decrease (−5.77%) among girls who spent 2 h at most with computers, but these were not significant changes, as shown in Table 5. 

### 3.4. Self-Rated Health

Self-rated health as a reliable measure of overall health showed a gender difference: significantly more boys reported their health to be excellent compared to girls both in the baseline (35.33% of boys vs. 21.85% of girls, *p* < 0.001) and follow-up surveys (40.35% of boys vs. 28.43% of girls, *p* = 0.023). The proportion of students who reported excellent health increased among boys by 5.02% and significantly increased in girls as well (6.58%, *p* = 0.04) via the follow-up survey compared to the baseline (Table 6).

**Figure 1:**
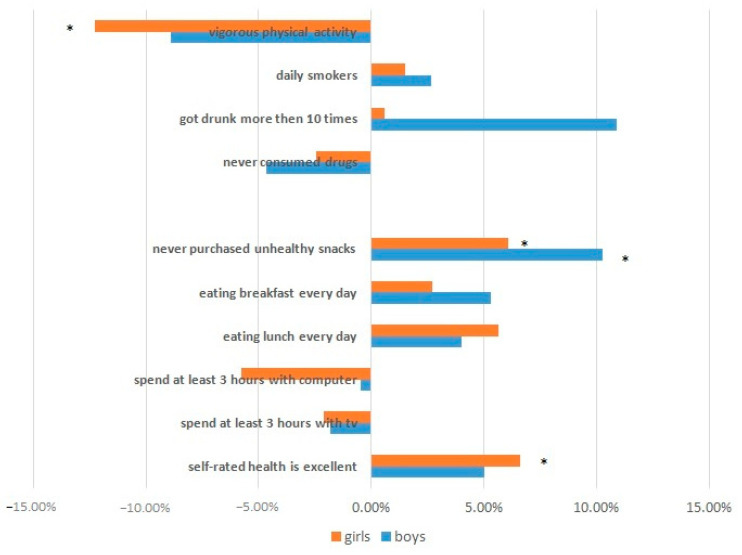
Summary of outcomes in self-rated health and health behaviour expressed as per cent change from baseline to follow up (* *p* < 0.05).

Changes in health behaviour are summarised in Figure 1 as a percent change in the specified variables. The upper four indicators show as unfavourable, and the rest of the indicators show favourable changes between the baseline and follow-up surveys. 

## 4. Discussion

Our paper describes the short-term evaluation of a health-promoting programme implemented in a high school in the second largest city of Hungary. Quantitative evaluation was used to compare the health and health behaviour of students before and 4 months after the intervention. The proportion of pupils who were in excellent health increased; significant improvements were shown in dietary habits such as never purchasing unhealthy snacks in school. However, the proportion of inactive pupils increased compared to the baseline survey.

The programme aimed at addressing all major dimensions of school health, including health education, the provision of daily physical education in the school for students, personal development, and conflict management among teachers. The programme was supported by the school leadership, which enabled the evaluation by ensuring the conditions for data collection, which was based on the validated scales of the Hungarian version of the HBSC questionnaire. 

Limitations derive from the lack of qualitative methods, as well as teachers and parents not being included due to limitations in finances, human resources, and time. The quantitative evaluation was limited to pupils in those three classes who received the intervention and were still in school at the time of the follow up (4 months after the end of the programme). 

A comparison of our results to those of others is limited by the fact that school health-promoting programmes show great diversity in terms of intervention design and methods, target groups, duration, financing, as well as evaluation design [19]. A health promotion programme similar to ours (implemented in one school for one academic year, using a pre–post-intervention comparison before and after) in Sweden [20] helped maintain adolescents’ very good or good sense of wellbeing, assessed via a 33-item scale similar to but not identical with subjective health assessed using a single item in our study. Another programme piloted in one Dutch high school for 4 years—using mixed methods of evaluation in repeated cross-sectional surveys—found significantly improved psychosocial health, but also—in contrast to our findings—reduced alcohol use, smoking, and sedentary time among students [11]. An Australian school-based health promotion programme of a seven-week duration for urban indigenous youth was also evaluated via a questionnaire survey delivered pre- and post-intervention [21], which found an improved frequency of breakfast similar to our finding, as well as an increased frequency of physical activity in contrast to our results.

Secondary school students in Canada were investigated in a study aimed to evaluate the impact of a web-based school nutrition intervention on eating behaviour traits, body weight, body size perception, and dissatisfaction. No significant negative changes were observed between the intervention and control groups for eating behaviour traits, body weight concern, body size perception, and dissatisfaction; however, the results suggest a trend for a positive effect of the intervention on susceptibility to hunger in boys [22]. Large-scale interventions implemented in several schools of a school district or at the national level provide more reliable evidence for effectiveness. A total of 67 such studies involving 1345 schools and a range of health issues were summarised in a systemic review which found positive effects for physical activity, fruit and vegetable intake, and tobacco use, among others, but no impact on alcohol and drug use [23]. The latter, that is, substance use not impacted by the intervention, is similar to our findings. However, the quality of evidence in these studies was deemed to be low-to-moderate in spite of the fact that all of the studies were cluster-randomised controlled trials, which are not the first choice of evaluation design in health promotion [8] and can only be designed at the level of geographical areas, not for single school interventions.

Health-promoting activities not having an impact on physical activity was an intriguing finding of a recent paper that compared health behaviour in 11–17year-old Israeli adolescents based on data from the 2018/2019 Health Behaviour in School-aged Children study in Israel, comparing youth in health-promoting and non-health-promoting schools [24].

Our findings on physical activity are in accordance with the results from HBSC surveys. The overall level of physical activity had been low and decreased with age among school-age children in the past two decades according to the HBSC data from 32 countries [25]. PA was shown to sharply decrease from childhood to adolescence (ages 6–19) across sex in the US National Health and Nutrition Examination Survey [26]. On the other hand, scientific evidence for the health benefits of physical activity in youth have accumulated since the early 2000s [27,28], so encouraging physical activity was aimed at in many early health-promoting projects, though initial recommendations called for it in a non-compulsory manner [29].

Our evaluation provided evidence that non-compulsory daily physical education cannot counter the secular effect of age on physical inactivity in adolescents. However, policy-level support for teachers to implement organised PA activities in schools can increase students’ PA, as it was shown in an Australian cluster-randomised trial [30].

Though the contribution of our findings to national policy cannot be proven, it is a fact that the Hungarian Act CXC on National Public Education mandated the gradual introduction of compulsory daily physical education in public schools for all students 6–18 years of age, which became nationwide when coupled with a complex fitness assessment from 2015 [31].

## Figures and Tables

**Table 1:** Comparison of students of years 9–12 in the intervention school with the Hungarian sample of the HBSC (years 5–11).

Demographic Indicators	HBSC 2010 ^a^	Intervention School 2011	*p*
Sample size	8096	944	
Grades	5, 7, 9, 11	9, 10, 11, 12	
Girls (%)	51.13	58.92	<0.001
Type of permanent residence (any type of city, %)	86.5	76.6	<0.001
Mother’s education (higher education, %)	21.8	51.1	<0.001
Father’s education (higher education, %)	16.6	41.9	<0.001
Computer ownership (two or more in family, %)	51.6	69.6	<0.001
Perceived family wealth			
--very well or quite well off, %	39.5	27.5	0.018
--average, %	54.6	62.2	
--not so or not at all well off, %	5.9	10.3	

^a^ Data are from the National Report of HBSC 2010 [17].

**Table 2:** Total number and gender distribution of students by grade and survey.

	**Grade 9**		**Grade 10**		**Grade 11**		**Grade 12**		**Total Surveyed**	
	total (N)	girls (%)	total (N)	girls (%)	total (N)	girls (%)	total (N)	girls (%)	total (N)	girls (%)
baseline survey	265	61.5	267	56.1	214	61.6	n.i. *	n.i.	746	59.5
follow-up survey	n.i.	n.i.	230	62.6	235	63.4	165	66.6	630	63.9

* n.i.: not included.

**Table 3:** Physical activity (total number and percentage of students by gender and survey time).

	**Boys**		**Girls**		**Boys**		**Girls**		***p* (Baseline and Follow-Up 1)**	
	**n**	**%**	**n**	**%**	**n**	**%**	**n**	**%**		
Prevalence of weekly activity										
four to seven times per week	108	35.88%	81	18.20%	73	32.02%	60	14.71%	boys	0.3371
less than four times a week	193	64.12%	364	81.80%	155	67.98%	348	85.29%	girls	0.2391
total	301	100.00%	445	100.00	228	100.00%	408	100.00%		
gender difference at baseline: *p* < 0.001, gender difference at follow up: *p* < 0.001										
Hours of weekly activity									*p* (baseline and follow-up 1)	
4–7 h per week	139	47.44%	111	25.52%	97	43.11%	88	22.00%	boys	0.3646
less than 4 h a week	154	52.56%	324	74.48%	128	56.89%	312	78.00%	girls	0.1769
total	293	100.00%	435	100.00%	225	100.00%	400	100.00%		
gender difference at baseline: *p* < 0.001, gender difference at follow up: *p* < 0.001										
Hours of school PE									*p* (baseline and follow-up 1)	
daily	12	4.00%	8	1.80%	7	3.07%	12	2.95%	boys	0.5395
not daily	288	96.00%	436	98.20%	221	96.93%	395	97.05%	girls	0.3486
total	300	100.00%	444	100.00%	228	100.00%	407	100.00%		
gender difference at baseline: *p* = 0.052, gender difference at follow up: *p* = 0.675										
Physical activity (composite variable)									*p* (baseline and follow-up 1)	
vigorous	72	64.86%	52	34.67%	47	55.95%	37	22.42%	boys	0.201
not vigorous	39	35.14%	98	65.33%	37	44.05%	128	77.58%	**girls**	**0.010**
total	111	100.00%	150	100.00%	84	100.00%	165			
gender difference at baseline: *p <* 0.001, gender difference at follow up: *p* < 0.001										

**Table 4:** Dietary habits (total number and distribution of students by gender and survey time).

	**Boys**		**Girls**		**Boys**		**Girls**		***p* (Baseline and Follow-Up 1)**	
	**n**	**%**	**n**	**%**	**n**	**%**	**n**	**%**		
Sweets and sugary soft drinks										
never	96	33.45%	98	22.58%	97	43.69%	114	28.64%	**boys**	**0.0111**
ever	191	66.55%	336	77.42%	125	56.31%	284	71.36%	**girls**	**0.0484**
total	287	100.00%	434	100.00%	222	100.00%	398	100.00%		
gender difference at baseline: *p* < 0.001, gender difference at follow up: *p* < 0.001										
Breakfast consumption on school days									*p* (baseline and follow-up 1)	
daily	167	55.67%	227	51.13%	139	60.96%	219	53.81%	boys	0.2484
not daily	133	44.33%	217	48.87%	89	39.04%	188	46.19%	girls	0.3814
total	300	100.00%	444	100.00%	228	100.00%	407	100.00%		
gender difference at baseline: *p* = 0.473, gender difference at follow up: *p* = 0.151										
Lunch consumption on school days									*p* (baseline and follow-up 1)	
daily	225	75.76%	271	61.04%	181	79.74%	270	66.67%	boys	0.2755
not daily	72	24.24%	173	38.96%	46	20.26%	135	33.33%	girls	0.0691
total	297	100.00%	444	100.00%	227	100.00%	405	100.00%		
gender difference at baseline: *p* < 0.001, gender difference at follow up: *p* = 0.001										

**Table 5:** Substance use, leisure time tv, and computer use (total number and distribution of students by gender and survey time).

	**Boys**		**Girls**		**Boys**		**Girls**		***p* (Baseline and Follow-Up)**	
	**n**	**%**	**n**	**%**	**n**	**%**	**n**	**%**		
Current use of tobacco										
non-smoker	247	82.06%	376	84.68%	183	80.97%	336	82.76%	boys	0.7695
smoker	54	17.94%	68	15.32%	43	19.03%	70	17.24%	girls	0.4264
total	301	100.00%	444	100.00%	226	100.00%	406	100.00%		
gender difference at baseline: *p* = 0.318, gender difference at follow up: *p* = 0.522										
Drunkenness during lifetime									*p* (baseline and follow-up)	
never	119	39.93%	225	50.90%	78	34.51%	190	46.80%	boys	0.2425
ever	179	60.07%	217	49.10%	148	65.49%	216	53.20%	girls	0.2444
total	298	100.00%	442	100.00%	226	100.00%	406	100.00%		
gender difference at baseline: *p* < 0.001, gender difference at follow up: *p* < 0.001										
Drug use during lifetime									*p* (baseline and follow-up)	
never	280	95.24%	402	93.93%	202	90.58%	359	91.58%	boys	0.0721
ever	14	4.76%	26	6.07%	21	9.42%	33	8.42%	girls	0.2608
total	294	100.00%	428	100.00%	223	100.00%	392	100.00%		
gender difference at baseline: *p* = 0.449, gender difference at follow up: *p* = 0.674										
Leisure time tv use									*p* (baseline and follow-up)	
<2 h	251	83.67%	379	85.75%	194	85.47%	355	87.86%	boys	0.7539
≥3 h	49	16.33%	63	14.25%	33	14.53%	49	12.14%	girls	0.3883
total	300	100.00%	442	100.00%	227	100.00%	404	100.00%		
gender difference at baseline: *p* = 0.032, gender difference at follow up: *p* = 0.456										
Leisure time computer use									*p* (baseline and follow-up)	
<2 h	210	70.01%	320	71.91%	160	70.49%	317	77.68%	boys	1.0000
≥3 h	90	29.99%	125	28.09%	67	29.51%	91	22.32%	girls	0.0436
total	300	100.00%	445	100.00%	227	100.00%	408	100.00%		
gender difference at baseline: *p* = 0.003, gender difference at follow up: *p* = 0004										

**Table 6:** Self-rated health (total number and distribution of students by gender and survey).

	**Boys**		**Girls**		**Boys**		**Girls**		***p*** **(Baseline and Follow-Up)**	
	**n**	**%**	**n**	**%**	**n**	**%**	**n**	**%**		
Self-rated health										
excellent	106	35.33%	97	21.85%	92	40.35%	116	28.43%	boys	0.2389
poor, satisfying, and good	194	64.67%	347	78.15%	136	59.65%	292	71.57%	**girls**	**0.0430**
total	300	100.00%	444	100.00%	228	100.00%	408	100.00%		
gender difference at baseline: *p* < 0.001, gender difference at follow up: *p* = 0.023										

## Data Availability

The data presented in this study are available on written request from the corresponding author. The data are not publicly available due to one paragraph of the ethical permission. Authorization of the request by the Data Protection Office of the University of Debrecen, Hungary is necessary as well.
